# Cortico-cerebellar functional connectivity and sequencing of movements in schizophrenia

**DOI:** 10.1186/1471-244X-12-17

**Published:** 2012-03-12

**Authors:** Tomas Kasparek, Jitka Rehulova, Milos Kerkovsky, Andrea Sprlakova, Marek Mechl, Michal Mikl

**Affiliations:** 1 Department of Psychiatry, University Hospital Brno and Masaryk University, Jihlavska 20, 625 00 Brno, Czech Republic; 2 Behavioral and Social Neuroscience Research Group, CEITEC - Central European Institute of Technology, Masaryk University, Kamenice 5, 625 00 Brno, Czech Republic; 3 Department of Radiology, University Hospital Brno and Masaryk University, Jihlavska 20, 625 00 Brno, Czech Republic; 4 Molecular and Functional Neuroimaging Research Group, CEITEC - Central European Institute of Technology, Masaryk University, Kamenice 5, 625 00 Brno, Czech Republic

**Keywords:** Schizophrenia, Motor cortex, Cerebellum, Connectivity, Movement sequencing, Neurological soft signs

## Abstract

**Background:**

Abnormal execution of several movements in a sequence is a frequent finding in schizophrenia. Successful performance of such motor acts requires correct integration of cortico-subcortical processes, particularly those related to cerebellar functions. Abnormal connectivity between cortical and cerebellar regions with resulting cognitive dysmetria has been proposed as the core dysfunction behind many signs and symptoms of schizophrenia. The aim of the present study was to assess if these proposed abnormalities in connectivity are a unifying feature of schizophrenia, or, rather, reflect a specific symptom domain of a heterogeneous disease. We predicted that abnormal functional connectivity between the motor cortex and cerebellum would be linked with abnormal performance of movement sequencing.

**Methods:**

We examined 24 schizophrenia patients (SCH) and 24 age-, sex-, and handedness-matched healthy controls (HC) using fMRI during a modified finger-tapping task. The ability to perform movement sequencing was tested using the Neurological Evaluation Scale (NES). The subjects were categorized into two groups, with (SQ+) and without (SQ-) movement sequencing abnormalities, according to the NES-SQ score. The effects of diagnosis and movement sequencing abnormalities on the functional connectivity parameters between the motor cortex and cerebellum (MC-CRBL) and the supplementary motor cortex and cerebellum (SMA-CRBL) activated during the motor task were analyzed.

**Results:**

We found no effect of diagnosis on the functional connectivity measures. There was, however, a significant effect on the SQ group: SQ + patients showed a lower level of MC-CRBL connectivity than SQ- patients and healthy controls. Moreover, the level of MC-CRBL and SMA-CRBL negatively correlated with the magnitude of NES-SQ abnormalities, but with no other NES domain.

**Conclusions:**

Abnormal cortico-cerebellar functional connectivity during the execution of a motor task is linked with movement sequencing abnormalities in schizophrenia, but not with the diagnosis of schizophrenia per se. It seems that specific patterns of inter-regional connectivity are linked with corresponding signs and symptoms of clinically heterogeneous conditions such as schizophrenia.

## Background

Schizophrenia is a heterogeneous mental illness with a variable clinical manifestation. Individual patients present with a variable pattern of symptoms, including abnormalities of higher integrative functions, such as perception, thinking, cognition, and, quite frequently, abnormalities of motor functioning. Abnormalities of the motor system in schizophrenia are related to the minor neurological changes that are traditionally referred to as neurological soft signs (NSS). They include problems with sequencing of movements (i.e., performance of several movements one by one in turn), and coordination of movements (i.e., performance of several movements simultaneously in time). Dysfunction of movement sequencing seems to be the prominent motor symptom in schizophrenia [1]. NSS are independent of the extrapyramidal adverse effects of antipsychotic drugs; i.e., they are not a result of the treatment [2,3], but a reflection of the basic neurobiology of the illness.

At the level of brain physiology, schizophrenia is linked with abnormalities of brain connectivity. Inefficient or misleading communication between functional brain areas has been postulated to be the core dysfunction behind the clinical manifestation of the illness. Friston and Frith stressed the importance of corticocortical connectivity-fronto-temporal connectivity-- - and coined the term 'dysconnection' [4]. Later, Andreasen et al. formed the theory of cognitive dysmetria as a basic cognitive dysfunction that arises from abnormal coordination in loops formed by the cortex, thalamus, and cerebellum, with a special focus on cerebellar functions in the fine timing of motor and cognitive events [5]. Abnormal connectivity in cortico-cerebellar loops would lead to disorganization of complex neurophysiological and mental processes, as seen in schizophrenia. Although a substantial body of evidence for abnormal brain functional connectivity in schizophrenia exists [6,7], no overall specific pattern of connectivity changes in schizophrenia has emerged. The heterogeneity of the findings reflects the clinical heterogeneity of the illness, where various phenotypes stem from different patterns of connectivity changes.

Although an abnormal performance of movement sequencing has been linked previously with anatomical changes of the brain [8-10], the traditional view of NSS pathogenesis stresses an inefficient cooperation between cortical and subcortical areas involved in complex motor functions [11]. The correct performance of a sequence of movements requires accurate timing of the execution of individual motor plans, which indicates the involvement of the cerebellum or cooperation between cortical and cerebellar areas [12]. Abnormal cortico-cerebellar connectivity would, therefore, lead to abnormalities of movement sequencing performance, as seen in schizophrenia. Functional brain connectivity in the context of movement sequencing abnormalities has not been analyzed before.

To test the hypothesis that abnormal functional connectivity between the motor cortex and cerebellum is linked with abnormal performance of movement sequencing rather than with the diagnosis of schizophrenia, we used a nontrivial sequential motor task during fMRI examination to activate the sensorimotor system of the brain in order to assess its functional connectivity and to analyze the correlates of cortico-cerebellar connectivity with the magnitude of motor sequencing abnormalities in patients with schizophrenia.

## Methods

### Subjects

We analyzed data from 24 schizophrenia patients and 24 age-, sex-, and handedness- (all subjects were right-handed) matched healthy controls. The diagnosis of schizophrenia was verified using the Mini-International Neuropsychiatric Interview (M.I.N.I.) [13]. All subjects were treated with atypical antipsychotics; the mean daily dose in chlorpromazine equivalents was 442 mg (SD 286). Details of the demographic and clinical parameters are given in Table 1. The exclusion criteria were drug dependence (based on the M.I.N.I.; subjects with a history of drug abuse, but not dependence, were included), neurological or somatic conditions affecting the structure or function of the brain, and contraindications for MRI examination. Two schizophrenia patients were not able to undergo the MRI examination and were not included in subsequent analyses.

**Table 1:** Demographic and behavioral characteristics

Group	**No**.	Age (SD)	Gender (M/F)	Abuse (%)	FH-SCH (%)	Education P/S/U (%)	Unemployment (%)	Marital status Si/Ma/Di (%)	BAS (%)	AIMS (%)	SAS (%)	NSS-T (%)	SI (%)	MC (%)	SQ (%)
HC	24	31.8 (9.2)	11/13	0*	0	1/12/11* (4.2/50/45.8)	2* (8.3)	18/5/1 (75/20.8/4.2)	0*	0	0**	1** (4.2)	0*	0	1** (4.2)
SCH	24	32.8 (9.7)	11/13	5 (21)	3 (13.6)	4/17/3 (16.7/70.8/12.5)	16 (69.6)	16/4/3 (69.6/17.4/13)	5 (20.8)	0	15 (62.5)	21 (87.5)	8 (33.3)	3 (12.5)	16 (66.7)
SQ+	7	36.9 (6.6)	3/4	0	1 (16.7)	1/5/1 (14.3/71.4/14.1)	6 (85.7)	4/1/1 (66.7/16.7/16.7)	2 (28.6)	0	5 (71.4)	7 (100)	1 (14.3)	2 (28.6)	7* (100)
SQ-	17	31.1 (10.4)	8/9	5 (29)	2 (12.5)	3/12/2 (17.7/70.6/11.8)	10 (62.5)	12/3/2 (70.6/17.6/11.8)	3 (17.7)	0	10 (58.8)	14 (82.4)	7 (41.2)	1 (4.2)	9 (52.9)

HC- healthy controls, SCH- schizophrenia patients, SQ + schizophrenia patients with marked movement sequencing abnormalities (SQ score > 2), SQ- schizophrenia patients without movement sequencing abnormalities; M- male, F- female; R- right, M- mixed, L- left; FH-SCH- family history of schizophrenia; P- primary, S- secondary, U- university education; Si-single, Ma- married, Di- divorced; BAS- Barnes Akathisia Scale; AIMS- Abnormal Involuntary Movements Scale, SAS- Simpson-Angus Scale; NSS-T- neurological soft signs total score, SI- sensory integration, MC- motor coordination, SQ- movement sequencing. Frequencies of subjects with at least one mild impairment in NES are given here. * p < 0.05, ** p < 0.001 - significance level for group comparisons (HC vs. SCH, SQ + vs. SQ-)

Healthy subjects were recruited from the community, local staff, and medical students. They were screened for Axis I psychiatric conditions using the M.I.N.I. Details of the demographic parameters are given in Table 1. The exclusion criteria were drug dependence, family history of Axis I psychiatric conditions, neurological or somatic conditions affecting the structure or function of the brain, and contraindications for MRI examination.

The study was approved by the local ethics committee (Ethical committee of the University Hospital Brno Bohunice) and all subjects signed an informed consent form.

### Behavioral examination

All subjects were examined using the Neurological Evaluation Scale (NES) [14] to assess the expression of abnormal movement sequencing (NSS-SQ subscale; we also examined other domains-abnormalities of motor coordination NSS-MC subscale, and sensory integration NSS-SI subscale-to compare the relative frequencies of individual domains of neurological abnormalities in our sample). The ability to perform several movements in time-a sequencing of movements-was assessed using the "fist-ring test" (rapid alternation between clenching the fist and forming a ring using the thumb and forefinger), "fist-edge-palm test" (tapping the desk using the fist, edge of the hand, and palm of the hand), "rhythm tapping test" (reproduction of several rhythms), and the "Ozeretski test" (both hands are placed on the table, one palm down, one palm up, and the subject is asked to simultaneously alternate the position of the hands). All the tests are performed separately using both hands. The performance is rated on a three-point scale: "0" - normal performance, "1" - mild impairment, "2" - marked impairment. The final score of the "Mmovement sequencing domain" is a sum of all test ratings from both hands.

Handedness was also assessed using the NES scale-it is a performance-based evaluation- with hand dominance described as right, left, or mixed.

The magnitude of extrapyramidal adverse effects of antipsychotic drugs was assessed using the Barnes Akathisia Scale (BAS), the Simpson-Angus Scale (SAS), and the Abnormal Involuntary Movement Scale (AIMS). These scales were used to check if the NSS were related to the medication effects, rather than to brain abnormalities.

### fMRI examination

All subjects underwent functional MRI examination in a Philips Achieva 1.5 T scanner (FFE EPI sequence, 33 axial slices, 80 × 80, in-plane resolution 2.85 × 2.85 mm, slice thickness 3.7 mm, TR = 3 s, TE 50 ms). The activation paradigm consisted of a sequential finger-tapping task (repetitive tapping of the four fingers against a board), a finger opposition task (repetitive tapping of all fingers together against a board; included to evaluate if typical activation of cortico-cerebellar motor regions exists during a simple motor task), and a motor rest condition. The conditions were performed in 30 s blocks, and repeated 4 times (resulting in 120 images/time-points). Before entering the scanner, subjects were trained to perform the movements at around 2 Hz. Subjects were visually monitored during the fMRI examination, and, if necessary, instructed through headphones.

### Image processing

**Figure 1:**
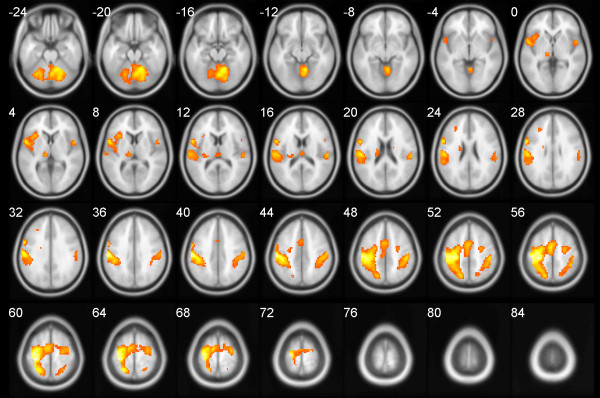
**Brain activation during sequential finger-tapping task**. Positive effect of condition (sequential finger-tapping task vs. rest) on the whole group level (p < 0.05 FWE corrected). The left side of the brain is on the left side of the image. Note that there were no significant differences in the brain activation between schizophrenia patients and healthy controls, nor were there any differences between the two schizophrenia subgroups.

The fMRI images were processed using the SPM8 toolbox (http://www.fil.ion.ucl.ac.uk/spm). They were realigned, co-registered to a high-resolution structural image, normalized to the standard MNI space, and smoothed using an 8 mm FWHM Gaussian kernel. The data entered the General Linear Model (GLM) design at a single-subject level as follows: two regressors of interest were created for the sequential movement condition and the simple opposite movement condition. The movement and rotations estimated during the realignment step were used to create six additional regressors of no interest. Contrast images for the condition where sequential tapping elicited higher activation compared to the motor rest condition entered second level analysis-a full factorial design with a fixed factor group (schizophrenia patients, healthy subjects). This GLM design was used for a) analysis of group differences in brain activation during sequential motor tasks (significance threshold p < 0.05, FWE corrected), and b) for creating a whole-sample activation map during sequential motor tasks (p < 0.05, FWE corrected). This whole-sample activation map was used to select coordinates in pre-defined seed regions for subsequent functional connectivity analysis (see Figure 1).

For the connectivity analysis, we extracted data from 5 mm spheres with centers at statistical local maxima in clusters in the left motor cortex MC (BA4, precentral gyrus, MNI oriae 3 -19 55), the suplmnaymtrcre M B6 N oriae 3-10 58), and the right cerebellum CRBL (anterior lobe, culmen, MNI coordinates 12-55 -23) from all single-subject data. The data were filtered (high-pass filter, AR), and adjusted for the effect of interest (contrast sequencing > rest, i.e., all other effects, including head motion parameters and mean, were filtered from the data). Subsequently, the first eigenvariate of the seed region BOLD time-course (i.e., the most typical representation of the time-course containing the largest part of the variability of the data) was extracted. The similarity of time-courses between individual seed regions was used as a measure of functional connectivity. To accomplish this, we performed a correlation analysis between the seed time-courses (MC-CRBL and SMA-CRBL) of every subject of the study. The significance of the correlations was tested using the one-sample *t*-test.

### Statistical analyses

The normality of the distribution of functional connectivity measures was tested using Kolmogorov-Smirnov test. The distribution did not differ significantly from the normal distribution; therefore, we used parametric tests for subsequent statistical analysis.

Group differences in demographic, behavioral, and clinical variables were tested using two-sample t-tests or chi-square tests. The relationships between continuous demographic, behavioral, and clinical variables were tested using correlation analysis.

The effect of diagnosis on the functional connectivity measures was tested using the two-sample *t*-test. The effect of SQ abnormalities on the functional connectivity measures was tested using correlation analysis between the SQ score and MC-CRBL and SMA-CRBL. We divided the schizophrenia patients according to the presence of marked movement sequencing abnormalities into two subgroups: patients with at least one marked abnormality in any SQ subtest (patients with a SQ score higher than 2) formed a group with sequencing abnormalities (SQ+), patients with at most mild abnormality in any SQ subtests (a SQ score less than or equal to 2) formed a group without sequencing abnormalities (SQ-). We specified ANCOVA designs with functional connectivity measures as a dependent variable, group (SQ+, SQ-, HC) as a categorical variable, and age as a continuous variable. The age parameter was included to account for the differences in age between the two patient subgroups and healthy controls group, since the age matching was performed only for the whole groups. After an omnibus test determined the significance of the effect of the group, we performed additional post-hoc least-significant-difference (LSD) testing of the within-group differences. Finally, we analyzed the confounding effects of demographic and clinical variables on connectivity parameters. If there were any significant relationships between these variables, we performed an analysis of covariance with the categorical parameter group (SQ + × SQ-) and the continuous parameter (the confounding variable) to assess its effect on group differences in connectivity. The level of significance was set to p < 0.05.

## Results

### Demographic data

There were no differences in age, gender, or marital status between the schizophrenia patients and healthy controls. Healthy controls had university education more frequently than schizophrenia patients (Chi-square 7.2, p < 0.05). Patients were also more frequently unemployed (Chi-square 18.6, p < 0.001). There were no differences in gender, age, education, employment, or marital status between the SQ + and SQ- patient groups.

### Behavioral and clinical data

Schizophrenia patients had higher frequencies of psychoactive drug abuse (1 patient with cannabis, 1 with alcohol, and 3 with polymorphic abuse including methamphetamine and cannabis) than healthy controls (Chi-square 5.4, p < 0.05). There was a trend toward a higher frequency of family history for schizophrenia in the schizophrenia group (Chi-square 3.5, p = 0.06). Schizophrenia patients had a positive BAS scale (Chi-square 5.6, p < 0.05) and SAS scale (Chi-square 21.8, p < 0.001) more often than healthy controls. Schizophrenia patients had significantly higher scores on the SAS scale (t = 47 < 0.001). No study subject had a positive AIMS score. No significant differences were found for the BAS score. A significantly higher proportion of schizophrenia patients had at least one mild impairment on the NSS scale (Chi-square 33.6, p < 0.001), NSS-SI subscale (Chi-square 9.6, p < 0.05), and NSS-SQ subscale (Chi-square 22.6, p < 0.001). There was a trend for a higher proportion of schizophrenia patients with at least one mild impairment of the NSS-MC subscale (Chi-quare 3.2, p = 0.07). Schizophrenia patients also had a higher total NSS score (t = 77 p < 0.001), NSS-SI score (t = 31 < 0.05), and NSS-SQ score (t = 47 < 0.001).

SQ + patients experienced more psychotic episodes (t = 27 < 0.05) than SQ- patients. There were no differences in diagnosis, duration of illness, antipsychotic medication, prescription of antiparkinsonian medication, rate of psychoactive drug abuse, or family history of schizophrenia. There were no differences in antipsychotic daily dose in chlorpromazine equivalents between the two patient groups, nor were there any significant differences in the magnitude of the SAS, AIMS, and BAS scores. SQ + patients had at least one impairment on the NSS-SQ (Chi-square 4.9, p < 0.05) subscale more frequently than SQ-patients. There were no differences in the expression of at least one mild impairment on the NSS total scale or the NSS-MC and NSS-SI subscales between the two groups. SQ + patients had a higher total NSS score (t = 28 < 0.05), and NSS-SQ subscale (t = 70 < 0.001). For frequencies, means, and standard deviations of demographic and clinical data, see Tables 1 and 2.

**Table 2:** Clinical characteristics

Group	Dg. SCH/SAF (%)	Duration	Episodes	Dose	AP MARTA/SDA(%)	BAS	AIMS	SAS	AntiPark (%)	NSS-T	SI	MC
SCH	21/3 (87.5/12.5)	9.2 (7.6)	4.2(2.0)	364.5 (203.1)	13/11 (54.2/45.8)	0.7 (1.9)	0	1.2 (1.3)	3 (12.5)	4.0 (2.4)	0.9 (1.4)	0.2 (0.5)
SQ+	5/2 (71.4/28.6)	13.8 (7.2)	5.7(1.3)*	360.6 (195.8)	4/3(57.1/42.9)	0.6 (1.1)	0	1.7 (1.5)	1 (14.3)	5.9* (2.2)	0.3 (0.8)	0.4 (0.8)
SQ-	16/1 (94.1/5.9)	7.8 (7.4)	3.6(1.9)	366.1 (212.0)	9/8 (52.9/47.1)	0.7 (2.2)	0	1.0 (1.1)	2 (11.8)	3.2 (2.1)	1.1 (1.5)	0.06 (0.2)

SCH- schizophrenia patients, SQ + schizophrenia patients with marked movement sequencing abnormalities (SQ score > 2), SQ- schizophrenia patients without movement sequencing abnormalities; SCH- schizophrenia, SAF- schizoaffective disorder; AP- antipsychotic medication, MARTA- multi-acting receptor-targeted antipsychotics, SDA- serotonin-dopamine antagonists; BAS- Barnes Akathisia Scale; AIMS- Abnormal Involuntary Movements Scale, SAS- Simpson-Angus Scale; AntiPark- antiparkinsonian medication; NSS-T- neurological soft signs total score, SI- sensory integration, MC- motor coordination, SQ- movements sequencing. * p < 0.05, ** p < 0.001 - significance level for group comparisons (SQ + vs. SQ-)

The NSS-SQ subscale did not correlate with the number of psychotic episodes, age, disease duration, or daily antipsychotic dose in chlorpromazine equivalents. There were also no significant correlations with BAS, AIMS, or SAS extrapyramidal scales. On the other hand, extrapyramidal symptoms measured using the SAS scale correlated significantly with the NSS-MC subscale (R = 0.47, p < 0.05).

### Functional connectivity

Brain activation patterns during the sequential finger-tapping task included sensorimotor cortical areas, prefrontal and cingular executive areas, the parietal and temporal supramodal cortex, thalamus, basal ganglia, and cerebellum; for details, see Figure 1 and Table 3. There were no significant differences in the activation pattern between schizophrenia patients and healthy controls, or between SQ + and SQ- patients.

**Table 3:** Brain activation during sequential finger-tapping task

Area	Brodmann Area	volume (cc) L/R	random effects: Max Value (x, y, z)
Precentral Gyrus	3, 4, 6, 44	12.8/3.1	14.5(-0 -24, 55)/8.2 (30, -10, 62)
Postcentral Gyrus	1, 2, 3, 5, 7, 40, 43	12.3/6.9	13.6(-3 -19, 38)/9.2 (45, -28, 47)
Paracentral Lobule	31	0.4/0.0	6.4(-,-19, 46)/-999.0 (0, 0, 0)
Superior Frontal Gyrus	6	1.7/1.1	9.1(-7 -9, 65)/7.9 (15, -4, 65)
Middle Frontal Gyrus	6	4.4/2.8	10.7(-7 -7, 57)/8.1 (24, -7, 51)
Inferior Frontal Gyrus	9, 44, 45, 47	2.4/0.1	9.9 (-56, 6, 25)/5.6 (53, 6, 25)
Medial Frontal Gyrus	6, 32	6.1/2.6	9.8(-6,-4, 54)/8.0 (3, 4, 50)
Cingulate Gyrus	24, 32	1.3/0.7	7.9(-3, 4, 45/. 3,4)
Insula	13, 40	5.2/0.8	8.7 (-48, -23, 19)/7.1 (50, -23, 19)
Superior Temporal Gyrus	22, 41, 42	3.3/1.0	8.2 (-50, 2, 1)/6.9 (50, 8, 1)
Transverse Temporal Gyrus	41, 42	1.4/0.3	7.8 (-56, -18, 13)/6.9 (53, -21, 13)
Superior Parietal Lobule	5, 7	3.5/0.9	10.5 (-21, -50, 61)/7.1 (24, -50, 59)
Inferior Parietal Lobule	2, 40	9.6/5.2	11.2 (-42, -31, 47)/8.2 (48, -28, 44)
Supramarginal Gyrus	40	0.5/0.0	7.4 (-42, -37, 36)/-999.0 (0, 0, 0)
Precuneus	7	3.6/0.9	8.6 (-18, -60, 51)/6.3 (18, -59, 54)
Lingual Gyrus	18	0.1/0.3	5.8 (-12, -83, -13)/6.9 (18, -77, -10)
Fusiform Gyrus	19	0.1/0.0	5.7 (-21, -80, -13)/-999.0 (0, 0, 0)
Lentiform Nucleus	*	0.1/0.0	5.2 (-30, -21, 5)/-999.0 (0, 0, 0)
Claustrum	*	0.1/0.0	5.8 (-27, 14, 9)/-999.0 (0, 0, 0)
Thalamus	*	1.3/0.2	7.3 (-12, -21, 5)/5.7 (6, -21, 13)
Declive	*	6.1/6.6	10.0 (-27, -60, -19)/10.9 (12, -54, -12)
Culmen	*	2.6/4.7	9.1 (-24, -57, -19)/10.4 (9, -54, -9)
Culmen of Vermis	*	0.2/0.1	8.8 (0, -65, -8)/9.3 (3, -63, -9)
Declive of Vermis	*	0.4/0.5	8.4 (0, -69, -11)/8.3 (3, -69, -13)
Cerebellar Lingual	*	0.3/0.7	5.6 (-3, -45, -12)/6.9 (9, -48, -14)

The mean of individual subject correlations between MC and CRBL (mean r = 0.23, SD 0.16, t = 9.9, p < 0.001) and SMA and CRBL (mean r = 0.26, SD 0.16, t = 11.0, p < 0.001) BOLD signals differed significantly from zero in the whole sample. There was a significant negative correlation between MC-CRBL and SQ score (r = 03, p < 0.05) and between SMA-CRBL and SQ score (r = 03, p < 0.05). No other NSS subscales correlated with the functional connectivity measures.

**Figure 2:**
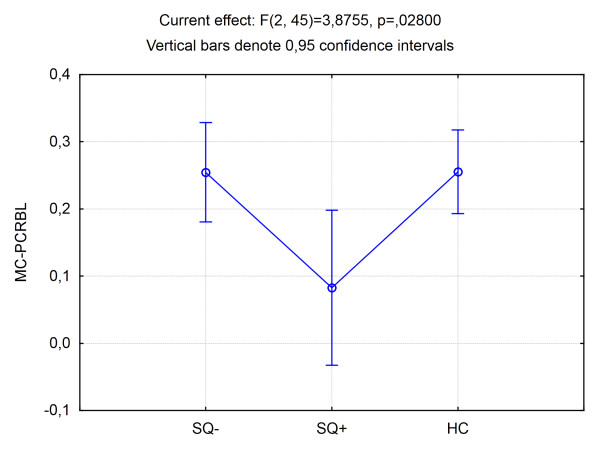
**Cortico-cerebellar functional connectivity - group differences**. Group differences in connectivity parameters between patients with (SQ+) and without (SQ-) marked movement sequencing abnormalities and healthy controls (HC) assessed using the ANCOVA design. MC-CRBL - correlation between motor cortex and cerebellar BOLD signal.

There was no effect of diagnosis on the functional connectivity measures: the whole group of schizophrenia patients did not differ from healthy controls. In the ANCOVA design there was a significant effect of SQ on the MC-CRBL functional connectivity measure (F = 3.4, p < 0.05). LSD post-hoc testing revealed significant differences between SQ + and SQ-patients and SQ + patients and healthy controls: SQ + patients had a lower magnitude of MC-CRBL correlations than both groups. No differences were present between SQ- patients and healthy controls (Figure 2). There was no significant effect on SMA-CRBL functional connectivity measures. 

Confounding effects: we found significant negative correlations between MC-CRBL and duration of illness (R = 03, p < 0.05). When using this variable as a covariate in a subsequent ANCOVA model with connectivity parameters as the dependent variable, there was still a significant effect of the SQ group on MC-CRBL (F = 7.2, p < 0.05). There was no effect of education on the magnitude of MC-CRBL and SMA-CRBL correlations (see the group differences in education between SCH and NC). There were also no significant correlations between daily antipsychotic dose (in chlorpromazine equivalents), MC-CRBL, and SMA-CRBL.

## Discussion

We found a significant effect of movement sequencing abnormalities on the functional connectivity between the motor cortex and cerebellum during the execution of sequential motor tasks in schizophrenia. We found a significant negative correlation between the magnitudes of movement sequencing abnormalities measured using the NES scale and the level of cortico-cerebellar connectivity: the more pronounced the motor abnormality, the less similar the BOLD signal between cortical and cerebellar regions. Only patients with impaired movement sequencing abilities had lower functional connectivity than healthy controls (in contrast to patients with unimpaired movement sequencing). This means that abnormal cooperation between the cerebellum and motor cortex during a motor task manifests as an impaired ability to sequence movements in time.

Several findings support the significance of cortico-eeelrfntoa dsoncin'fr various signs and symptoms of schizophrenia. Wilson et al. found right cerebellar dysfunction in a MEG study that analyzed cortico-cerebellar functional connectivity during tactile stimulation of the fingers that resulted in synchronous activation of the postcentral cortex and cerebellum in early onset psychosis [15]. Impaired functional connectivity between the cerebellum and the medial part of the superior frontal gyrus was observed during attentional tasks in schizophrenia [16]. Abnormal functional connectivity between the medial prefrontal cortex and the contralateral cerebellum was also found during the Hayling Sentence Completion Test in a high-risk sample [17], and in children and adolescents with schizophrenia during verbal working-memory tasks [18]. There are reports of abnormal functional connectivity between the cerebellum and cortex even at rest: parameters of resting state functional connectivity between the frontal cortex and cerebellum had the biggest discriminative value from a set of several regions for classification of schizophrenia patients and healthy controls [19]. Structural equation modeling allowed the analysis of the connectivity parameters between individual nodes of a network formed by the prefrontal cortex, thalamus, and cerebellum during an n-back working-memory task in schizophrenia: patients showed decreased cortico-cerebellar and thalamo-cerebellar functional connectivity, but increased thalamo-cortical functional connectivity [20].

Impaired functional cortico-cerebellar connectivity might result from abnormalities of some node of the cortico-subcortico-cerebellar circuits. However, several lines of evidence indicate that cerebellar dysfunction might have a key role in cortico-cerebellar dysconnection' and corresponding signs and symptoms, including movement sequencing abnormalities. Transcranial magnetic stimulation (TMS) data show that cerebellar activation or inhibition has a direct impact on the functioning of the motor cortex [21]. This finding, with the theoretical implications of the involvement of cerebellum in the pathogenesis of schizophrenia, provides further support for the validity of the regions we selected for functional connectivity analysis. Furthermore, there is evidence that sequence processing is one of the key functions of the cerebellum [12]. There are many findings that show cerebellar abnormalities in schizophrenia. Cerebellar inhibition of the motor cortex assessed by TMS is decreased in schizophrenia [22]. Reduction of cerebellar-dependent delay eyeblink conditioning [23] reflects impaired cerebellar time processing in schizophrenia. Cerebellar morphological changes are present in schizophrenia [24]; they are linked with NSS [25-27], including repetitive motor acts, i.e., sequences of movements [25,28,29]. Cerebellar abnormalities are linked to non-motor symptoms of schizophrenia, such as cognitive dysfunction [16,30,31]. Changes in cerebellar tract integrity that correlate with cognitive dysfunction [32] have been described in schizophrenia [33-35].

We were not able to find any effect of movement sequencing abnormalities on the pattern or magnitude of brain activation during the modified finger-tapping task. A previous fMRI study, using a similar behavioral paradigm, found reduced activation of the pallidum and putamen [36]. Our data show that impaired movement sequencing abilities are linked with brain connectivity, rather than with the magnitude of regional activation, which points to the original concept of NSS as a consequence of impaired cooperation between system subcomponents, and not a single localized pathology [11]. On the other hand, the lack of group differences in brain activation patterns may also reflect an inadequate behavioral paradigm for movement sequencing examination. Although the principle behind the modified sequential finger-tapping task is similar to the NES scale sequencing tasks, the tasks are not identical and may require different effort for their execution and reflect different brain loads. Our paradigm was, however, adequate for the analysis of brain connectivity in the context of movement sequences, which was the primary focus of our study.

The impaired ability to sequence movements was the most frequent movement abnormality in schizophrenia patients: 67% of the patients had at least one mild impairment of movement sequencing as compared to a 33% incidence of sensory integration problems, and 13% of motor coordination abnormalities. The pattern and incidence of individual NSS categories are in accordance with previous findings [1]. The relative predominance of movement sequencing abnormalities in schizophrenia might reflect its link to a proposed neurobiological substrate of the illness-brain 'dysconnection'.

Patients with marked movement sequencing impairment had more psychotic episodes than those without movement sequencing disturbances. Moreover, cortico-cerebellar functional connectivity correlated negatively with the duration of treatment. These findings may mean that there is a progressive worsening of cortico-cerebellar connectivity with a corresponding impairment of movement sequencing as a result of consecutive psychotic outbreaks. Our study was, however, a cross-sectional comparison. Therefore, we cannot exclude the possibility that patients with marked impairments of movement sequencing abnormalities had such marked impairment present from the beginning of the illness. Previous studies show that the expression of NSS at the time of the first episode of schizophrenia predicts a worse response to treatment and worse outcomes [37-39]. Abnormalities of cerebellar morphology are also linked with poor outcomes [40]. The fact that there was a still a significant effect of movement sequencing abnormalities present in the post-hoc analysis of covariance suggests that movement sequencing abnormalities and their link to cortico-cerebellar connectivity are not only the result of illness progression.

Several factors might limit our results. The most important are the effects of antipsychotics, extrapyramidal symptoms, and abuse of psychoactive substances. The severity of movement sequencing abnormalities does not correlate with antipsychotic treatment parameters, nor with the severity of extrapyramidal symptoms. Similarly, the differences in cortico-cerebellar connectivity between the two patient groups are not a result of antipsychotic treatment or extrapyramidal symptoms. This is supported by previous findings [2,3]. These factors are, however, linked significantly with the other NSS cluster-movement coordination abnormalities-which may reflect different neurobiology and etiology of individual movement abnormalities in schizophrenia. Findings have shown a relationship between the severity of cerebellar impairment and co-morbid alcohol abuse in schizophrenia [41]. In our study, however, there were no differences in co-morbid psychoactive substance abuse between the two patient groups. Finally, we assessed only the functional connectivity related to movement sequencing. But the cerebellum is involved in several other functions, including cognitive and affective ones [42]. Based on our study, we cannot exclude the possibility that there is a functional dysconnection of different parts of the cortex and cerebellum during the cerebellar functions other than movement sequencing in SQ- patients.

## Conclusion

Abnormal cortico-cerebellar functional connectivity during the performance of a motor task is linked with movement sequencing abnormalities in schizophrenia, but not with the diagnosis of schizophrenia per se. It seems that specific patterns of interregional connectivity are linked with corresponding signs and symptoms of clinically heterogeneous conditions such as schizophrenia.

## Competing interests

The authors declare that they have no competing interests.

## Authors' contributions

Author Tomas Kasparek designed the study, wrote the protocol, undertook the data analysis, and wrote the first draft of the manuscript. Author Jitka Rehulova managed the literature searches and contributed to data collection and preparation. Author Milos Kerkovsky contributed to the design of the study and its protocol. Authors Andrea Sprlakova and Marek Mechl were responsible for clinical interpretation and preprocessing of the MRI data. Author Michal Mikl contributed to the connectivity analysis. All authors contributed to and have approved the final manuscript.

## Pre-publication history

The pre-publication history for this paper can be accessed here:

http://www.biomedcentral.com/1471-244X/12/17/prepub
